# Population‐based Bayesian regularization for microstructural diffusion MRI with NODDIDA

**DOI:** 10.1002/mrm.27831

**Published:** 2019-05-26

**Authors:** Meghdoot Mozumder, Jose M. Pozo, Santiago Coelho, Alejandro F. Frangi

**Affiliations:** ^1^ Centre for Computational Imaging & Simulation Technologies in Biomedicine (CISTIB), Department of Electronic and Electrical Engineering The University of Sheffield Sheffield United Kingdom; ^2^ Department of Applied Physics University of Eastern Finland Kuopio Finland; ^3^ Centre for Computational Imaging & Simulation Technologies in Biomedicine (CISTIB), School of Computing and School of Medicine University of Leeds Leeds United Kingdom; ^4^ LICAMM Leeds Institute of Cardiac and Metabolic Medicine, School of Medicine University of Leeds Leeds United Kingdom

**Keywords:** biophysical tissue models, diffusion MRI, microstructure imaging, modeling, parameter estimation

## Abstract

**Purpose:**

Information on the brain microstructure can be probed by Diffusion Magnetic Resonance Imaging (dMRI). Neurite Orientation Dispersion and Density Imaging with Diffusivities Assessment (NODDIDA) is one of the simplest microstructural model proposed. However, the estimation of the NODDIDA parameters from clinically plausible dMRI acquisition is ill‐posed, and different parameter sets can describe the same measurements equally well. A few approaches to resolve this problem focused on developing better optimization strategies for this non‐convex optimization. However, this fundamentally does not resolve ill‐posedness. This article introduces a Bayesian estimation framework, which is regularized through knowledge from an extensive dMRI measurement set on a population of healthy adults (henceforth *population‐based prior*).

**Methods:**

We reformulate the problem as a Bayesian maximum a posteriori estimation, which includes as a special case previous approach using non‐informative uniform priors. A population‐based prior is estimated from 35 subjects of the MGH Adult Diffusion data (Human Connectome Project), acquired with an extensive acquisition protocol including high *b*‐values. The accuracy and robustness of different approaches with and without the population‐based prior is tested on subsets of the MGH dataset, and an independent dataset from a clinically comparable scanner, with only clinically plausible dMRI measurements.

**Results:**

The population‐based prior produced substantially more accurate and robust parameter estimates, compared to the conventional uniform priors, for clinically feasible protocols, without introducing any evident bias.

**Conclusions:**

The use of the proposed Bayesian population‐based prior can lead to clinically feasible and robust estimation of NODDIDA parameters without changing the acquisition protocol.

## 1 INTRODUCTION

Diffusion magnetic resonance imaging (dMRI) allows in vivo and noninvasive mapping of water molecules’ diffusive movement in biological tissues. This motion is constrained by the tissue microarchitecture.^1^ Hence, combined with biophysical modeling, dMRI is potentially capable of capturing microstructural features related to tissue constituents. There exists several modeling techniques in the literature capable of capturing such information. *Signal models*, for instance, directly model the dMRI signal with a particular functional form. The most common of them is the diffusion tensor imaging,^2^ which, despite its simplicity, can still provide meaningful biomarkers that are widely used as indications of microstructural tissue changes.^3^ *Micro‐structural models*,^4^ instead, derive the dMRI signal from a physical model of the tissue microstructure (e.g.^5^, ^6^, ^7^). This allows capturing more specific information of individual tissue constituents.

One of the most popular dMRI microstructural models is the neurite orientation dispersion and density imaging (NODDI).^7^ NODDI describes the signal generated from a voxel as arising from three independent non‐exchanging compartments: intra‐neurite, extra‐neurite, and cerebrospinal fluid (CSF). The intra‐neurite compartment is modeled as a set of sticks, i.e. cylinders with zero radius, the extracellular compartment is modeled as set of cylindrically symmetric diffusion tensors, and the CSF as an isotropic compartment. Instead of estimating all model parameters directly from the data, NODDI makes some assumptions and constrains a few of its microstructural parameters for estimating the rest. These assumptions have been shown^8^, ^9^, ^10^ to be not always valid and to result in biased estimates for the remaining model parameters.

**Figure 1:**
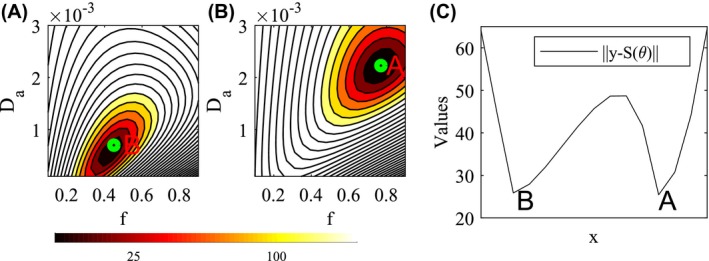
Illustration of the degeneracy present in the likelihood function of the dMRI signal for the NODDIDA model. From the example NODDIDA parameters, ***θ***, in the Set B of Jelescu et al^11^, we generated the dMRI measurements, **y**, for a clinically feasible protocol and SNR. The residual norm, ‖**y**‐**S**(**θ**)‖, presents 2 local minima with very similar values: the parameter point, **A**, generating the measurements, and another spurious minimum, **B**. Plots (A) and (B) display the cellular fraction, *f*, and intracellular diffusivity, $D_{a}$, plane (fixing the rest of the parameters) including the parameter points **B** and **A**, respectively. Plot (C) displays the line connecting both local minima

Jelescu et al^11^ suggested an alternative approach where these microstructural parameters were no longer considered fixed or constrained, called NODDIDA (NODDI with Diffusivity Assessment). This approach removes the incorrect assumptions of NODDI, but it makes the problem ill‐posed^10^: i.e. multiple parameter sets can describe the dMRI signal equally well (see Figure 1) and hence the solution is not unique. Also, it reduces NODDI from a three‐compartment model to a two‐compartment model, by eliminating the isotropic compartment. Hence, the model is only applicable in brain regions with minimal CSF contamination.

Despite these difficulties, these models describe several useful microstructural parameters for detecting pathological conditions, meriting further investigation. For example, the neurite orientation distribution has demonstrated potential in capturing white matter disarray in Alzheimer's disease (AD).^12^ Extracellular radial diffusivity increases with reduced myelination,^13^, ^14^ and is observed in the development of multiple sclerosis (MS) and AD. Since demyelination, unlike axonal loss, is in many cases reversible, a method that differentiates between the two has potential clinical value. NODDIDA parameters are also prospective biomarkers for other brain disorders, such as axonal loss in MS and AD could be inferred from the intra‐neurite fraction,^13^ accumulation of fluids in intra‐ and extracellular spaces in cerebral edema and beading could be inferred from the intra‐ and extra‐neurite diffusivites.^15^, ^16^

The ill‐posed parameter estimation in NODDIDA, sometimes referred to as *degeneracy*, requires some consideration. The NODDIDA degeneracy has been sometimes attributed to the non‐convexity of the problem resulting in multiple local minima of the cost function. Non‐convexity can be tackled using repeated local optimizations, starting from random parameter initializations, and selecting the solution with global minimum residual error.^11^ But this strategy does not address the ill‐posedness. The method produced reasonable estimates with extensive dMRI measurement protocols. However, for clinically feasible dMRI measurements, the method fails to resolve the NODDIDA degeneracy since there are multiple global minima with equal cost function value (as illustrated in Figure 1), making the optimum identification unstable for any noise level.

It was observed in Jelescu et al^11^ for a sample of cases, that the actual solution presented a wider basin of attraction in the parameter space than the spurious one. Assuming this observation is valid in general, they suggested to consider a corresponding alternative criterion for estimating the optimal solution: from several random initializations, selecting the local minimum with higher prevalence. This method was subsequently applied for a microstructural model similar to NODDIDA in Novikov et al.^10^ However, this assumption is not correct in all situations. The case illustrated in Figure 1 is a counterexample, showing larger basin around the wrong minima. A systematic comparison of these methods, has not yet been performed.

A machine learning Bayesian approach, based on training using simulated data and assuming the traces of the intra‐ and extra‐axonal diffusivities to be similar, has been proposed in Reisert et al.^17^ The approach provided largely unreliable estimates for extracellular diffusivities, putting in doubt its assumption. In addition, this method estimates the parameters expected value, ignoring (not solving) the possible bimodality of the posterior. Moreover, the implementation of methods factoring out the neurite orientation distributions^10^, ^17^ is not straightforward, limiting its applicability.

In this paper, we demonstrate for the first time, that parameter estimation approaches proposed earlier^10^, ^11^ can be described within the Bayesian estimation formalism as maximum a posteriori (MAP) estimation with uniform probability density function (pdf) priors on the parameters. Subsequently, we used a prior on model parameters by estimating their probability density function from extensive dMRI measurements in a population of 35 healthy adult subjects. We coin these *population‐based priors*, since they were estimated from a parameter sample in a reference population. We then compared MAP estimates with uniform and population‐based priors using common optimization methods, using real dMRI data. We also compared these approaches to NODDI. We demonstrate that the estimation using the proposed population‐based prior provides most reliable parameter estimates on clinical data that can be implemented on clinical dMRI protocols. However, no clear difference is observed between the performance of previously published optimization methods for each prior model. The proposed method is easily implemented by a straightforward modification of NODDI toolbox, increasing its potential application in clinical diagnosis.

## 2 METHODS

### 2.1 dMRI data

We have used the Massachusetts General Hospital (MGH) Adult Diffusion data,^18^ available from the Human Connectome Project (HCP).^19^ It is a high resolution, high *b*‐value dMRI dataset, obtained from 35 healthy adult subjects between 20 and 59 years old, using a protocol that used a substantially large set of *b*‐values and gradient directions (compared to clinical measurement protocols). Each subject of the MGH HCP has 40 $b_{0}$ images and measurements from 64 diffusion directions for $b\;=\;1000,\;3000\;{\text{s/mm}}^{2}$, 128 directions for $b\;=\;5000\;{\text{s/mm}}^{2}$, and 256 directions for $b\;=\;10\;000\;{\text{s/mm}}^{2}$.

We also used an independent dataset acquired by a 3T Siemens MAGNETOM ${\text{Prisma}}^{\mathrm{Fit}}$ system with 80 mT/m maximum gradient strength, which is comparable to a clinical scanner. An EPI/spin echo (SE) diffusion‐weighted pulse sequence was used with 75 ms echo time, 2700 ms pulse repetition time, and a 128 × 128 acquisition matrix resulting in an isotropic voxel size of 2.5 mm. 11 *b*‐values were acquired (*b* = 0, 250, 350, 450, 550, 650, 750, 850, 1150, 1500, 2000 ${\text{s/mm}}^{2}$) with 12 $b_{0}$ images and 60 non‐co‐linear magnetic field gradient directions for the others. The total acquisition time was 33 minutes.

Only voxels with minimal CSF contamination were used in the analysis, selected by a threshold of 1% CSF fraction estimated by the “free water elimination” technique in Pasternak et al.^20^

### 2.2 NODDIDA model

The NODDIDA microstructural model^10^, ^11^, ^21^ considers a distribution of axonal fibers with two tissue components resulting in a biexponential kernel,

(1) $$K\left(b,\;\xi \right)=f\;e^{-b\;D_{a}\xi^{2}}\;+\;\left(1-f\right)\;e^{-bD_{e}^{⊥}-b\left(D_{e}^{\|}-D_{e}^{⊥}\right)\xi^{2}},$$

dependent on the gradient strength, *b*, and the projection, $\xi \;=\;\hat{g}\cdot \hat{n}$, of the gradient direction, $\hat{g}$, into the fiber direction, $\hat{n}$. This kernel describes the diffusion due to a single axon and its extracellular matrix. Here, *f* describes the intracellular (axonal) fraction, $D_{a}$ is the intracellular diffusivity, and $D_{e}^{\|}$ and $D_{e}^{⊥}$ are the parallel and perpendicular extracellular diffusivities. The dMRI signal attenuation is given by the convolution of the kernel with the fiber orientation distribution function $p(\hat{n})$:

(2) $$S\equiv S_{\hat{g}}\left(b\right)=S_{0}\cdot \int d\hat{n}\;p\left(\hat{n}\right)\;K\left(b,\hat{g}\cdot \hat{n}\right).$$

NODDI^7^ and NODDIDA models consider the Watson spherical distribution, $p(\hat{n}|\kappa ,\;\hat{\mu })$, as fiber orientation distribution, parametrized by its main orientation $\hat{\mu }$ and the concentration *κ*, characterizing its anisotropy. Let $\theta \;=\;(f,\;D_{a},\;D_{e}^{\|},\;D_{e}^{⊥},\;\kappa )$ denote the NODIDDA model parameters, and let ***θ*** ↦ **S**(***θ***) be the deterministic forward model defined by (2). By deterministic, we mean, the model **S** does not contain any signal noise or model inaccuracies. Typical clinical dMRI scanners show SNRs >3 for the human brain,^22^ and as such the additive Rician noise, ϵ, in measurements can be approximated by a Gaussian distribution.^23^ Thus, the actual dMRI measurements can be modeled as

(3) $$y=S\left(\theta \right)+\epsilon ,\;\epsilon ∼N\left(0,\;\Gamma_{\epsilon }\right).$$

so that their conditional probability is

(4) $$\pi \left(y|\theta \right)=N(S\left(\theta \right),\;\Gamma_{\epsilon }).$$

Assuming independent noise of the same standard deviation for all measurements, the covariance matrix will be proportional to the identity matrix, $\Gamma_{\epsilon }\;=\;\sigma^{2}I$.

### 2.3 Bayesian estimation

In the Bayesian approach to inverse problems, all unknowns and measured quantities are considered random variables and the uncertainty of their values is encoded into a probability density function (model). Using the Bayes theorem, we can express the posterior distribution

(5) π(θ|y)∝π(y|θ)π(θ)

in terms of the measurements model, *π*(**y**|***θ***), and the prior pdf on the model parameters, *π*(***θ***). All these pdfs are probability densities on some high‐dimensional space. One standard criterion for the estimation of the model parameters from the posterior probability, is the *maximum a posteriori* (MAP) estimate.

If a uniform prior distribution is considered for the model parameters, θ∼U(a,b), and the Gaussian measurement model (4) is explicitly expanded, the MAP estimate is given by

(6) $$\begin{array}{cc} \theta_{\mathrm{MAP}} & =\text{arg}\max_{\theta }\pi \left(\theta |y\right)=\text{arg}\max_{\theta }\;\exp(-\frac{1}{2}{\left\|y-S\left(\theta \right)\right\|}_{\Gamma_{\epsilon }^{-1}}^{2})\;U\left(a,b\right)\\ & =\text{arg}\min_{\theta }{\left\|y-S\left(\theta \right)\right\|}^{2},\;\text{subject to}\;\;\theta \in \left[a,b\right]. \end{array}$$

Thus, this leads us to the least square cost function typically used^10^, ^11^, ^21^ with box constraints

(7) $$f\in \left[0,\;1\right]\subset R,\;D_{a},\;D_{e}^{\|},\;D_{e}^{⊥}\in \left[0,\;4\right]\subset R,\;\kappa \in \left[0,\;64\right]\subset R.$$

This estimator can be easily implemented as a modification to the NODDI toolbox^7^, ^11^ and is available for download from Mozumder.^24^

This cost function is usually interpreted as a maximum likelihood estimation (MLE) subject to constraints.^11^ However, the Bayesian formulation allows us to consider diverse priors, incorporating available information on the problem at hand via the pdf of the model parameters.

### 2.4 Population‐based priors

We use informative priors estimated from a sample of dMRI datasets with extended diffusion protocols. We used horizontal midbrain dMRI slices from the 35 subjects from the MGH HCP database. In contrast to more common clinical dMRI protocols, this protocol used to acquire the MGH HCP, had a substantially larger set of *b*‐values and gradient directions. This makes the parameter estimation problem better posed, and the larger number of directional measures increases accuracy.^10^, ^11^ We considered the method outlined in Jelescu et al^11^ for solving Equation (6) using several parameter initializations and choosing the solution with minimum residual errors. The obtained parameters have been considered as ground truth (GT). A total of *N* = 44 931 horizontal midbrain voxels have been analyzed, obtaining the corresponding *N* sets of NODDIDA parameters:

(8) $$\{\theta^{\left(l\right)},\;l=1\ldots N\}.$$

**Figure 2:**
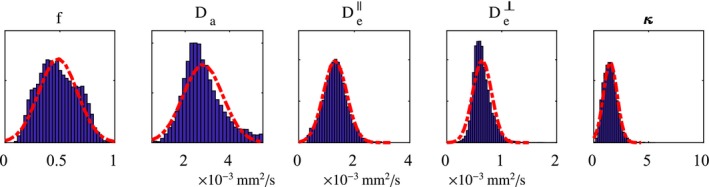
Distribution of NODDIDA parameters in the population estimated from midbrain sections of 35 patients from the MGH HCP dataset. They have been approximated by a multivariate Gaussian distributions (red dashed lines), defining the population‐based prior. Their cross‐correlations are not shown here

The parameter distribution in this sample (shown in Figure 2) provides an estimate of their population pdf, which is used as prior in MAP estimation. All parameters display near‐symmetric unimodal pdfs. Thus, we model them jointly as a multidimensional Gaussian pdf,

(9) $$\pi \left(\theta \right)=N\left(\bar{\theta },\;\Gamma_{\theta }\right),$$

which allows an easy treatment of their correlation, with the mean parameter vector, $\bar{\theta }$, and covariance matrix, $\Gamma_{\theta }$, estimated from the sample (8). Mean and covariance calculated from the 35 subjects are provided in the Supporting Information Equation S1. Proceeding as in Equation (6), but using this Gaussian prior, the MAP estimate is given by

(10) $$\begin{array}{cc} \theta_{\mathrm{MAP}} & =\text{arg}\max_{\theta }\exp(-\frac{1}{2}{\left\|y-S\left(\theta \right)\right\|}_{\Gamma_{\epsilon }^{-1}}^{2}-\frac{1}{2}{\left\|\theta -\bar{\theta }\right\|}_{\Gamma_{\theta }^{-1}}^{2})\\ & =\arg\min_{\theta }{(\|y-S\left(\theta \right)\|}_{\Gamma_{\epsilon }^{-1}}^{2}+{\left\|\theta -\bar{\theta }\right\|}_{\Gamma_{\theta }^{-1}}^{2}), \end{array}$$

which is a generalized least mean square cost function.

The covariance $\Gamma_{\epsilon }$ depends on the noise of the dMRI experimental data. We estimated it from the value of $S_{0}$ and assumed a SNR = 50. This SNR was arbitrarily chosen, since typical clinically feasible protocols does not acquire many $S_{0}$ images,^11^ and hence, the SNR cannot be directly estimated from the standard deviation of $S_{0}$ signals.

### 2.5 Optimization strategies

The calculation of the maximum posterior estimate, $\theta_{\mathrm{MAP}}$, with both uniform (6) and population‐based Gaussian (10) priors, requires solving a nonlinear optimization problem and concomitant iterative optimization. We have used the Levenberg‐Marquardt method, following the same approach as in Jelescu et al.^11^ The MAP estimations with each type of prior, was carried out using three different optimization strategies:

#### 2.5.1 Single random initialization

The optimization algorithm is run only once, considering randomly sampled initialization parameters in the interval (7). This strategy only guarantees the localization of a random local optimum. Thus, the instability of the result evidences the non‐convexity of the optimization problem.

#### 2.5.2 Maximum a posteriori selection

The optimization algorithm is run multiple times with different initializations.^11^ The estimate yielding the maximum a posteriori probability is selected as estimate of the global optimum. We have considered 20 initializations, the same number as in Novikov et al.^10^ This allows its direct comparison with their results, and we have observed no significant difference using a larger number of initializations.

#### 2.5.3 Highest prevalence selection

Optimization from multiple initializations. The most common solution is considered as the estimate representing the local optimum with wider basin of attraction.^10^ As for the previous strategy, 20 initializations were used.

These three optimization strategies, combined with the two pdfs, give a total of six estimation strategies. For completeness, we have also compared the estimates obtained via NODDI (as in the NODDI toolbox^7^, ^11^). NODDI fixes two diffusivities ($D_{a}\;=\;D_{e}^{\|}\;=\;1.7\;\times \;10^{-3}\;{\text{mm}}^{2}$/s) and assumes that the third diffusivity is constrained ($D_{e}^{⊥}\;=\;\left(1-f\right)D_{e}^{\|}$). Hence, it estimates only *f*, *κ* out of the five NODDIDA parameters. Table 1 lists the seven estimation strategies with the corresponding acronyms used in this work.

**Table 1:** Acronyms for the models and estimation methods considered in this paper

Acronym	Prior	Optimization strategies
N	Uniform	NODDI
U1	Uniform	Single initialization.
Um	Uniform	Maximum a posteriori (20 init.)
Up	Uniform	Highest prevalence (20 init.)
G1	Population‐based	Single initialization.
Gm	Population‐based	Maximum a posteriori (20 init.)
Gp	Population‐based	Highest prevalence (20 init.)

### 2.6 Experiments

From the complete dMRI extended acquisition protocol of the MGH HCP dataset, we considered a subset emulating a clinically feasible protocol.^11^ This subset included 3 *b*‐values ($b\;=\;0,\;1000,\;3000\;{\text{s/mm}}^{2}$), consisting of 1 $b_{0}$ image and 30 non‐co‐linear gradient directions for other shells. The $b\;=\;3000\;{\text{s/mm}}^{2}$ is the closest value to $b\;=\;2000\;{\text{s/mm}}^{2}$, used in Jelescu et al,^11^ available in the MGH HCP database. Despite this increased *b*‐value, it is still clinically feasible.^25^

The parameters estimated from the complete extended protocol were used for two purposes. First, the population‐based prior, *π*(***θ***), was estimated from them. Second, they were considered as the GT for the evaluation of the seven approaches considered, when applied on the clinically feasible subset. For a fair comparison, for the analysis of each subject, the prior in methods G1, Gm, and Gp did not include information from the same subject. That is, the prior estimation and accuracy evaluation were performed in a leave‐one‐subject‐out fashion.

#### 2.6.1 Evidencing the degeneracy

In order to illustrate the presence of a degenerated estimation in NODDIDA parameter and to explore in detail how this affects different estimation strategies, we considered two random voxels selected one from the corpus callosum (CC) and another from the posterior limb of the internal capsule (PLIC), from subject *MGH_1001*.

#### 2.6.2 Brain maps illustration

Two different brain regions of subjects *MGH_1001* and *MGH_1002* were considered to explore the spatial continuity of the model parameters and the importance of the generated noise with each of the seven estimation strategies. The NODDIDA parameter maps using the clinically feasible subset were also compared to the GT.

#### 2.6.3 Global accuracy evaluation

The midbrain slices of the full set of 35 subjects of the MGH HCP database was considered to evaluate and compare the accuracy of the seven estimation strategies when applied to the clinically feasible subset. To further explore the impact of the noise level on the parameter estimation accuracy, we subdivided the set of voxels into three groups, according to the SNR calculated using the standard deviation of the $S_{0}$ data: SNR < 25, 25 ≤ SNR ≤ 35, and 35 < SNR. The estimation accuracy for each of the parameters, $\theta \;=\;(f,\;D_{a},\;D_{e}^{\|},\;D_{e}^{⊥},\;\kappa )$, was measured by the relative error

(11) $$E_{\alpha }=\left|\theta_{\alpha }^{\left(\mathrm{Estimated}\right)}-\theta_{\alpha }^{\left(\mathrm{GT}\right)}\right|/\theta_{\alpha }^{\left(\mathrm{GT}\right)}\times 100\%,\;\text{for}\;\alpha =1,\;\ldots ,\;5.$$

#### 2.6.4 Using an independent dataset

We used the independent dMRI dataset from the MAGNETOM Prisma${}^{\mathrm{Fit}}$ system to test the performance of the seven estimation strategies. First, the whole dataset (all *b*‐values and gradient directions) were used to estimate the GT parameters. Then, a clinically feasible subset with three *b*‐values (b = 0, 1150, 2000 ${\text{s/mm}}^{2}$), with 1 $b_{0}$ image and 30 directions from other shells were used for the evaluation of the estimation strategies. The population‐based prior used in this case was based on the MGH HCP data.

## 3 RESULTS

### 3.1 Evidencing degeneracy

**Figure 3:**
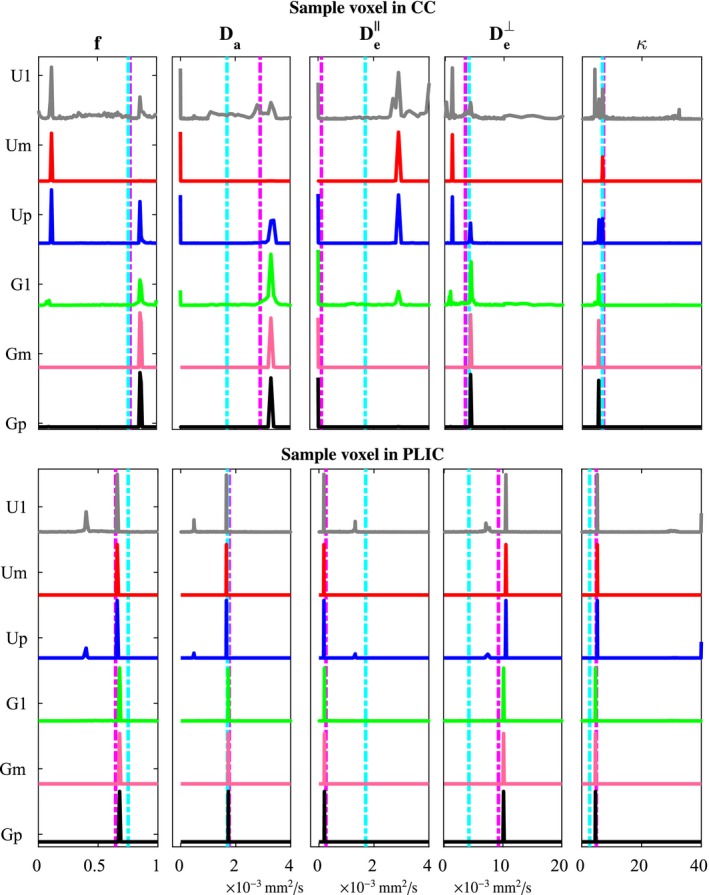
Illustration of the degeneracy in NODDIDA parameter estimation in two random voxels from CC and PLIC. The estimation of each of the NODDIDA parameters from a clinically feasible subset of dMRI measurements using the different estimation methods produce histogram of solutions, which can be multimodal. The cyan dotted lines represent the estimates using NODDI. The parameter estimates using the full extended protocol (GT) is shown in magenta dotted lines

Figure 3 displays the histogram of estimates, for each model parameters obtained with each estimation method. It presents separately the results for a random voxel of CC and for a random voxel of PLIC. The multimodal distribution shown by U1 evidences the non‐convexity of the dMRI likelihood for NODIDDA, presenting many local optima. The results for G1, show that this non‐convexity is already partially mitigated for the posterior with the population‐based prior. The global optimum selected by Um is close to the GT for the PLIC voxel, but identifies the wrong value in the CC. Only a slight improvement is obtained by the higher prevalence criteria in Up. In contrast, the population‐based prior allows both Gm and Gp to localize the correct optimum. We can also observe that the values assumed by NODDI for the diffusivities are suboptimal, thus biasing the estimates for *f* and *κ*, in agreement with,^8^ especially in the PLIC.

### 3.2 Brain dMRI example

**Figure 4:**
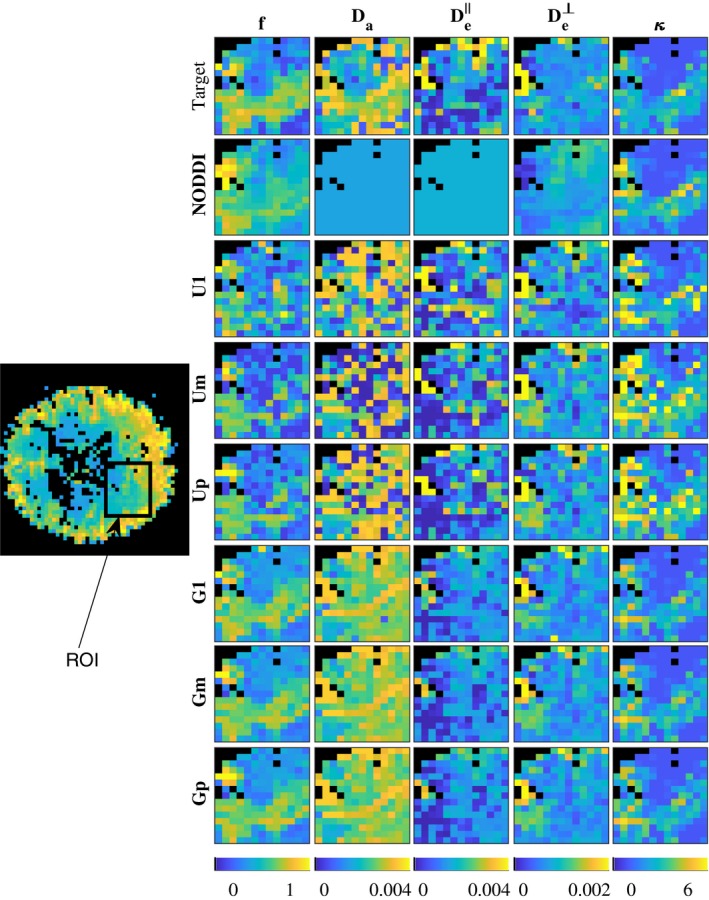
Map of NODDIDA parameters estimated from a brain region of interest (ROI) from subject *MGH_1001* (indicated within a rectangle in its $S_{0}$ image on the left). Top row show the GT parameters estimated using the whole set of extended dMRI measurements. The rows below show the results from the seven different methods applied on a clinically feasible subset of measurements

**Figure 5:**
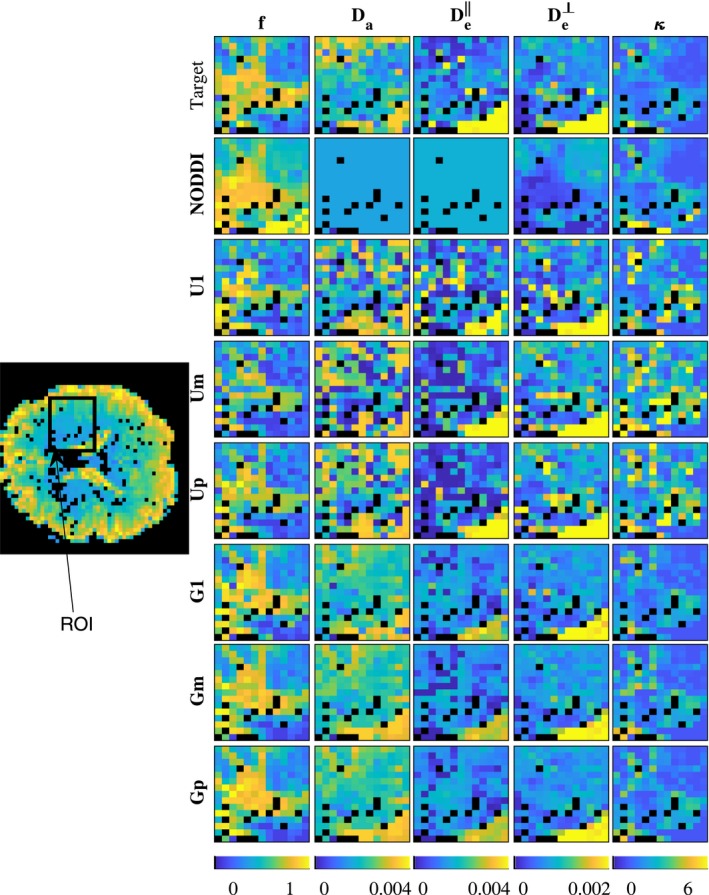
Map of NODDIDA parameters estimated from a brain region of interest (ROI) from subject *MGH_1002* (indicated within a rectangle in its $S_{0}$ image on the left). Top row show the GT parameters estimated using the whole set of extended dMRI measurements. The rows below show the results from the seven different methods applied on a clinically feasible subset of measurements

Figures 4 and 5 show model parameter maps in two different regions of the brain, each from a different subject. Parameter map of an entire brain slice is displayed in Supporting Information Figure S1. The estimates with U1, Um, and Up are very noisy. In contrast, the estimates obtained with population‐based priors (methods G1, Gp, and Gm) are smoother and present more realistic patterns alike GT maps. In addition, the obtained values are in the ranges expected for human brains, qualitatively matching parameter maps in Novikov et al,^10^ obtained with more extensive dMRI acquisition protocols, and showing higher $\left(f,\;D_{a},\;\kappa \right)$ and lower $\left(D_{e}^{\|},\;D_{e}^{⊥}\right)$ in white matter compared to gray matter. The maps obtained by NODDI are also smooth and qualitatively similar for *f* and *κ*, but cannot estimate the diffusivities.

### 3.3 Evaluating global accuracy

**Figure 6:**
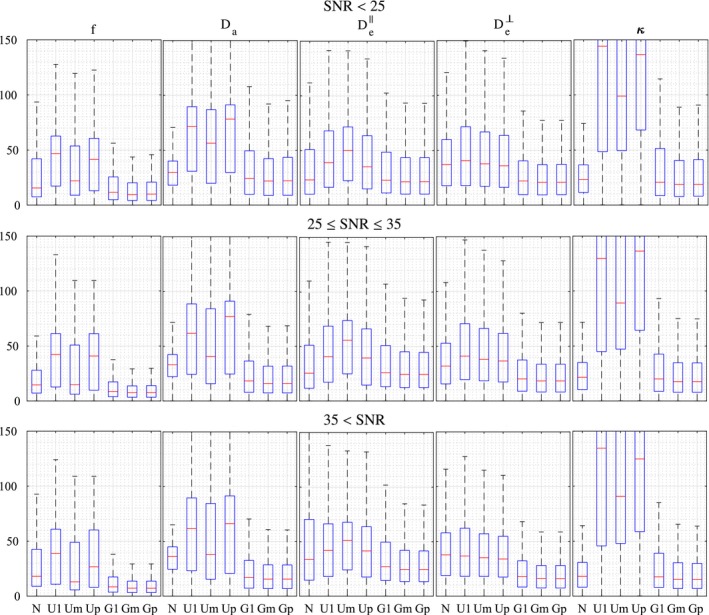
Accuracy of the estimation of the NODDIDA parameters by each of the estimation methods applied to a clinically feasible subset of measurements. The boxplots represent the distribution of the relative estimation error for each NODDIDA parameter and for each method. The results have been stratified in three groups of SNR ranges

Figure 6 displays the distribution of relative errors obtained using each of the seven estimation strategies, for different SNR ranges. The proposed population‐based priors gave lower errors for all parameters and all SNRs, for the three optimization strategies. Both highest prevalence (Gp) and minimum cost (Gm) displayed similar errors. The only slightly higher errors obtained with a single initialization (G1) suggest, in agreement with Figure 3, that the non‐convexity of the problem is largely mitigated. This could indicate that the global optima could be robustly found with fewer initializations than the 20 used here for Gm and Gp, making the estimation faster.

For the uniform prior, the maximum prevalence criterion (Up), provides in general worse results than minimum cost (Um). This contrasts with the observations in Jelescu et al,^11^ subsequently applied in Novikov et al,^10^ suggesting that minimum cost is a better criterion for the estimation of NODDIDA parameters.

The errors provided by NODDI were smaller than the ones from the NODDIDA estimation with uniform prior for all parameters. This happens even for the diffusivities, which have given fixed values in NODDI. In contrast, the inclusion of the population‐based prior leads to smaller errors also in comparison to NODDI.

The errors in the group with higher SNR are, in general, smaller. But this effect is more prominent for estimates with population‐based priors. Thus, Gm estimations could further benefit from increased dMRI signal quality. The estimation with uniform priors is apparently not improved by the increase in SNR, probably due to the presence of bimodality in the estimations.

### 3.4 Independent dataset

**Figure 7:**
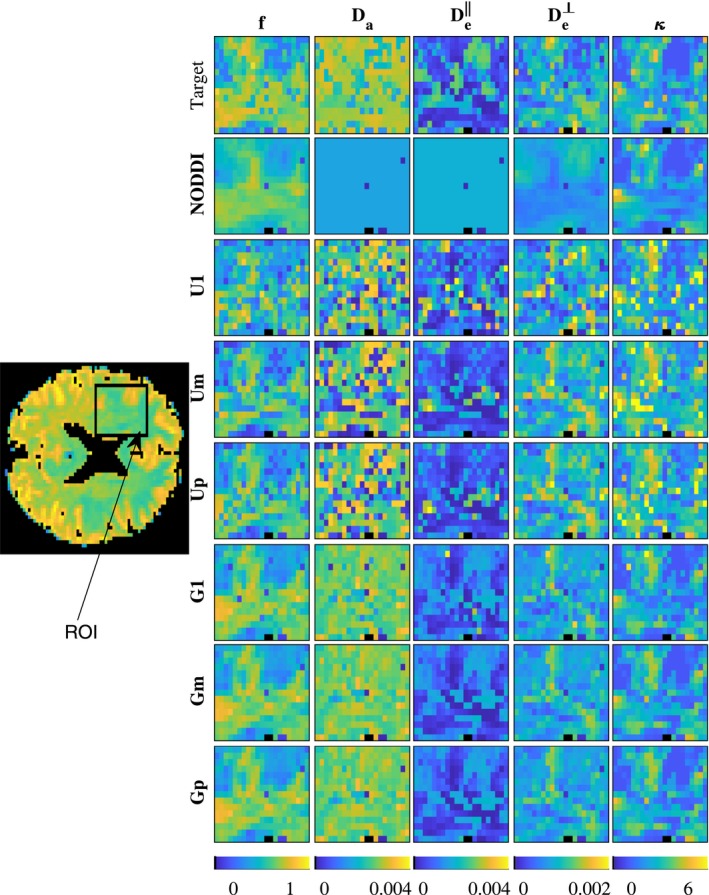
Map of NODDIDA parameters estimated from a brain region of interest (ROI) from the independent dMRI dataset (indicated within a rectangle in its $S_{0}$ image on the left). Top row show the GT parameters estimated using the whole set of extended dMRI measurements. The rows below show the results from the seven different methods applied on a clinically feasible subset of measurements

**Figure 8:**
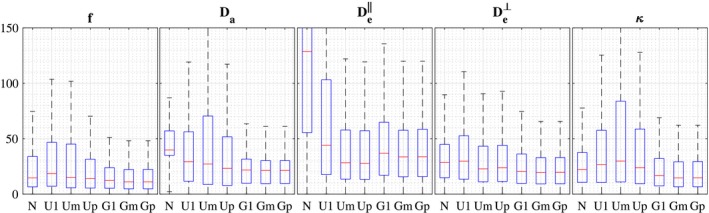
Accuracy of the estimation of the NODDIDA parameters by each of the estimation methods applied to a clinically feasible subset of measurements from the independent dMRI dataset. The boxplots represent the distribution of the relative estimation error for each NODDIDA parameter and for each method

Figure 7 displays parameter maps from an independent dMRI dataset. We observe that the proposed population‐based prior still provide smooth realistic parameter maps, closer to the target parameter maps. Figure 8 displays the distribution of relative errors obtained using the seven estimation strategies on the whole brain slice. The proposed population‐based priors gave lower errors for all parameters.

## 4 DISCUSSION

This work addresses parameter estimation in microstructural and in general, multi‐compartmental dMRI models. Estimation of parameters in multi‐compartmental dMRI models, such as NODDIDA, is inherently ill‐posed. This problem fundamentally arises when microstructural parameter values for different compartments are exchanged resulting in indistinguishable dMRI signals. Approaches to resolve this degeneracy have focused on developing better optimization strategies addressing non‐convex optimization. This, however, does not solve the ill‐posed nature of the problem for clinically relevant dMRI acquisition protocols and SNR, where acquiring extensive diffussion orientations is unfeasible. The use of priors in the estimation of NODDIDA parameters has been suggested earlier in Jelescu et al.^11^ However, its use has been ill‐advised in Novikov et al,^10^ following the prevalent belief among the diffusion community that priors are equivalent to constraints introducing bias in parameter estimation.

Nevertheless, we demonstrate in this work that the use of population‐based priors can substantially increase the robustness and accuracy of the NODDIDA parameters estimates without introducing noticeable bias. The mean estimation errors reduced to below 16% using the proposed prior, compared to below 42% using previously proposed priors. There are two aspects to highlight here. First, in contrast with NODDI constraints, the prior distribution can model parameter correlations without fixing them to exact parameter values. Second, prior information is encoded based on the observation of the model parameter distribution across distinct patient populations, not in hypothetical parameter values.

In our methods we chose SNR 50, since clinically feasible datasets doesn't have multiple $b_{0}$ values to allow the estimation of SNRs. A good guess of SNR can further improve the estimation accuracy, see Supporting Information Figure S2 and the discussion therein.

We evidence that previously proposed approaches in Novikov et al^10^ and Jelescu et al^11^ are akin to using non‐informative uniform priors within our Bayesian estimation framework. To develop more informative priors integrating information from specific populations, we considered the distribution of the model parameters estimated from 35 subjects of the MGH dataset from the HCP.^19^ These data comprise dMRI measurements with an extensive protocol including high *b*‐values, for which the degeneracy issue is minimal. The resulting parameters distribution was sufficiently well approximated by a multivariate Gaussian. The use of a Gaussian prior leads also to Gaussian posterior, convenient for computational optimization.

We tested previously proposed estimation approaches using uniform and population‐based priors in experimental datasets with clinically achievable dMRI acquisition protocols. For this, we selected appropriate subsets of measurements from the MGH dataset. This allowed evaluating the accuracy of the estimations from clinically feasible acquisitions, considering the model parameters estimated from the extensive protocol as GT. Observe that, unlike the previous NODDI/NODDIDA studies,^7^, ^10^, ^11^, ^17^ the evaluation was carried out against GT values calculated from real experimental data.

First, we explored in two sample voxels the behavior of the estimations for each method. Second, we qualitatively investigated the smoothness and plausibility of the spatial map of estimated parameters in two ROIs from two subjects. Next, we quantified the global accuracy of the estimations by the distribution of the relative errors in the dataset of 35 subjects. Finally, we used an independent dMRI measurement from a clinically comparable scanner to evaluate the estimation accuracies. Consistently across the four experiments, the introduction of the population‐based priors, effectively deals with the ill‐posedness of the problem and produce substantially more accurate results than previous techniques.

The multimodality of the parameter estimates using a single parameter initialization with the uniform prior (U1), observed in Figure 3, evidences the non‐convexity of the problem, as earlier reported in Jelescu et al.^11^ The known, more severe, ill‐posedness of the problem is evidenced in the instability of the global optimum with minimum residual (Um). In contrast with the observation and suggestion in Novikov et al^10^ and Jelescu et al,^11^ the selection of the wider optimum by the higher prevalence criterion (Up) does not solve the problem. This is further evidenced in the noisy parameter maps (Figures 4 and 5) obtained from any of the three criteria with uniform prior. Further, the largest errors observed (Figure 6) for the higher prevalence criteria (Up) discourages the use of this criteria. A few more examples are presented in Supporting Information Figures S3‐S6.

The non‐convexity of the estimation problem is largely reduced by the introduction of the population‐based prior, as evidenced in the removing of most of the multimodality in Figure 3 for a single initialization (G1). This is further reflected in the significant error reduction (Figures 6 and 8) for all methods including the population‐based prior (G1, Gm, and Gp) but with only slightly larger errors for G1, indicating that the search for the global optimum is much simplified.

Figure 6 points out the impact on the estimation accuracy of the level of noise in the three SNR ranges considered is negligible for NODDI and for the methods with uniform prior. In contrast, the gain in parameter accuracy with the quality of the signal is clear for the methods with the population‐based prior. This can be interpreted considering that the existing bimodality in the likelihood function is a persistent obstruction to the accuracy even with infinitesimal noise, but the removal of the bimodality by the population‐based prior releases the achievable accuracy from this obstruction.

NODDI provides accuracies (Figure 6) similar to the best method (Gm) for the dispersion parameter, *κ*, and, to some extent, for the diffusivities, $D_{a}$ and $D_{e}^{\|}$, especially for measurements with low SNR. However, NODDI presents substantially larger errors for the cellular fraction, *f*, and cannot estimate the variations in the diffusivities, since, in contrast with NODDIDA, they are fixed to predefined values (see Figures 4, 5 and 7).

The population‐based prior in this work was based on measurements from the Connectome scanner which has very high 300 mT/m maximum gradient strength, compared to clinical scanners which have 40‐60 mT/m maximum gradient strength. Since the observed diffusion values in dMRI depends partially on the diffusion gradient strengths and other scanning parameters,^26^ the presented prior might cause some bias in the estimation of the extra and intracellular diffusion coefficients, when applied to data obtained with other scanners or protocols. The presented experiment on an independent dMRI data acquired with a clinical scanner, demonstrates that the use of the prior still results in the same reduction of the ill‐posedness. However, since no ground truth is available in this case, we cannot discard a possible bias.

In the context of disease detection and treatment, changes in NODDIDA parameters ($\kappa ,\;D_{e}^{⊥}$) have been reported^12^, ^13^, ^14^ in multiple sclerosis and Alzheimer's disease, and is known to be potentially useful for several other disorders such as beading, edema, and inflammation.^10^ The parameter variations of $D_{a},\;D_{e}^{\|}$, not accessible by NODDI, are also potentially useful in detection of edema and inflammation. Figure 6, shows how utilizing the conventional uniform prior model or NODDI would lead to large estimation errors and could make estimation of NODDIDA parameter changes unfeasible in clinical settings. Using the population‐based prior, the errors are decreased in all parameters, for all SNR ranges. This decrease is particularly marked in $D_{a},\;\kappa$ and *f*, with mean errors below 16% in the more clinically relevant parameters ($D_{a},\;D_{e}^{⊥},\;\kappa ,\;f$) for SNR ≥35, indicating the potential of the use of population‐based prior in robust NODDIDA parameter estimation and subsequent clinical diagnosis development.

In this work, we have estimated the prior from a population of healthy subjects. For clinical applications investigating any pathology, a more general prior should be estimated from a population including also cases with such pathologies. We expect this will be feasible in the future, when extensive acquisition protocol‐based dMRI data from pathology cases will be available. We plan to construct such general priors in the future, based on pathology cases.

## 5 CONCLUSIONS

In this work, we introduced a novel framework of NODDIDA estimation, which allowed the use of a population‐based prior information in a Bayesian formulation. The prior was estimated from the distribution of NODDIDA parameters obtained from the publicly accessible MGH HCP dataset^19^ with dMRI from 35 subjects with an exceptionally extensive acquisition protocol. The parameter distribution was approximated by a multivariate Gaussian distribution defining the prior. This leads also to a Gaussian posterior, whose optimization is easily implemented as a modification of the NODDI toolbox. The code used for estimation can be downloaded from Mozumder^24^ and used in conjunction to the NODDI toolbox.

The results indicate that, contrary to previous claims,^10^ the use of priors within the Bayesian estimation framework, can lead to accurate and robust estimation of the NODDIDA parameters. The integration of the population‐based prior, with the minimum cost criteria to find the global optimum, provides parameter estimation accuracies surpassing the ones from previous methods. The results also suggest that this prior largely removes the ill‐posedness, and even partially the non‐convexity of the estimation problem. This method provides mean relative errors below 16% for the more clinically relevant parameters ($D_{a},\;D_{e}^{⊥},\;\kappa ,\;f$) using clinically feasible SNRs and datasets. The proposed method can be potentially developed for clinical diagnosis of brain disorders using the estimated NODDIDA parameters.

## Supporting information


**FIGURE S1** Map of NODDIDA parameters estimated from a midbrain slice from subject *MGH_1001*. Top row show the GT parameters estimated using the whole set of extended dMRI measurements. The rows below show the results from the seven different methods applied on a clinically feasible subset of measurements
**EQUATION S1** The mean ***θ*** of the NODDIDA model parameters $\theta \;=\;(f,\;D_{a},\;D_{e}^{\|},\;D_{e}^{⊥},\;\kappa )$, calculated from midbrain slices of the 35 subjects of MGH HCP database
**FIGURE S2** Accuracy of the estimation of the NODDIDA parameters by each of the previously proposed estimation methods (N, U1, Um, Up) and G1, Gm, Gp assuming SNR 50, assuming SNR 20, and using their true (calculated) SNRs. The boxplots represent the distribution of the relative estimation error for each NODDIDA parameter and for each method, with a clinically feasible subset of measurements. We observe a slight improvement in the estimation of $\kappa ,\;D_{e}^{\|}$ and $D_{e}^{⊥}$, using the proposed methods, when SNR 20 or the estimated SNRs were used. This is because, the mean SNR from all the MGH HCP data was around 35, and hence choosing SNR 20 was a closer approximation to the true SNR, than choosing SNR 50
**FIGURE S3** Estimates from a voxel of corona radiata with clinical subset of measurements. The cyan dotted lines represent the estimates using NODDI. The parameter estimates using the full extended protocol (GT) is shown in magenta dotted lines
**FIGURE S4** Estimates from a voxel of corona radiata with a different subset of measurements. The cyan dotted lines represent the estimates using NODDI. The parameter estimates using the full extended protocol (GT) is shown in magenta dotted lines
**FIGURE S5** Estimates from a gray matter voxel with clinical subset of measurements. The cyan dotted lines represent the estimates using NODDI. The parameter estimates using the full extended protocol (GT) is shown in magenta dotted lines
**FIGURE S6** Estimates from a different gray matter voxel with clinical subset of measurements. The cyan dotted lines represent the estimates using NODDI. The parameter estimates using the full extended protocol (GT) is shown in magenta dotted lines
