# Supplementary material for: Population‐based Bayesian regularization for microstructural diffusion MRI with NODDIDA
Source: Magn Reson Med. 2019 May 26;82(4):1553–65. doi: 10.1002/mrm.27831 (PMC6771666; doi:10.1002/mrm.27831)
Supplement: Supplementary file 1 — FIGURE S1 Map of NODDIDA parameters estimated from a midbrain slice from subject MGH_1001. Top row show the GT parameters estimated using the whole set of extended dMRI measurements. The rows below show the results from the seven different methods applied on a clinically feasible subset of measurements EQUATION S1 The mean θ of the NODDIDA model parameters θ=(f,Da,De‖,De⊥,κ), calculated from midbrain slices of the 35 subjects of MGH HCP database FIGURE S2 Accuracy of the estimation of the NODDIDA parameters by each of the previously proposed estimation methods (N, U1, Um, Up) and G1, Gm, Gp assuming SNR 50, assuming SNR 20, and using their true (calculated) SNRs. The boxplots represent the distribution of the relative estimation error for each NODDIDA parameter and for each method, with a clinically feasible subset of measurements. We observe a slight improvement in the estimation of κ,De‖ and De⊥, using the proposed methods, when SNR 20 or the estimated SNRs were used. This is because, the mean SNR from all the MGH HCP data was around 35, and hence choosing SNR 20 was a closer approximation to the true SNR, than choosing SNR 50 FIGURE S3 Estimates from a voxel of corona radiata with clinical subset of measurements. The cyan dotted lines represent the estimates using NODDI. The parameter estimates using the full extended protocol (GT) is shown in magenta dotted lines FIGURE S4 Estimates from a voxel of corona radiata with a different subset of measurements. The cyan dotted lines represent the estimates using NODDI. The parameter estimates using the full extended protocol (GT) is shown in magenta dotted lines FIGURE S5 Estimates from a gray matter voxel with clinical subset of measurements. The cyan dotted lines represent the estimates using NODDI. The parameter estimates using the full extended protocol (GT) is shown in magenta dotted lines FIGURE S6 Estimates from a different gray matter voxel with clinical subset of measurements. The cyan dotted lines represent th [file MRM-82-1553-s001.pdf]

Supporting data

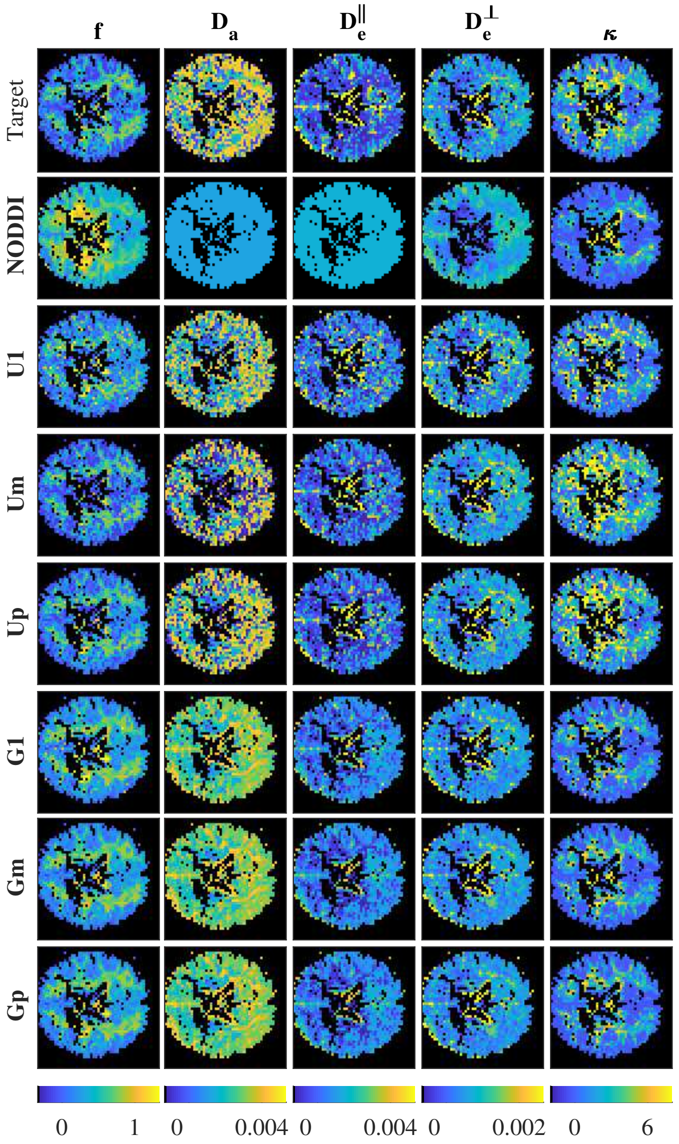

Supporting Information Figure S1: Map of NODDIDA parameters estimated from a mid brain slice from subject *MGH_1001*. Top row show the GT parameters estimated using the whole set of extended dMRI measurements. The rows below show the results from the 7 different methods applied on a clinically feasible subset of measurements.

Supporting Information Equation S1: The mean $\bar{\boldsymbol{\theta}}$ and the covariance matrix $\boldsymbol{\Gamma}_{\boldsymbol{\theta}}$ of the NODDIDA model parameters $\boldsymbol{\theta} = (f, D_\mathrm{a}, D_\mathrm{e}^{\|}, D_\mathrm{e}^{\perp}, \kappa)$, calculated from mid-brain slices of the 35 subjects of MGH HCP database

$$\bar{\boldsymbol{\theta}} = (0.48, 2.8 \times 10^{-3}\mathrm{s/mm}^2, 1.3 \times 10^{-3}\mathrm{s/mm}^2, 0.6 \times 10^{-3}\mathrm{s/mm}^2, 0.48),$$

$$\boldsymbol{\Gamma}_{\boldsymbol{\theta}} = \begin{bmatrix} 0.03 & 1.8 \times 10^{-5} & 1.8 \times 10^{-5} & 3.0 \times 10^{-6} & 0.05 \\ 1.8 \times 10^{-5} & 7.7 \times 10^{-5} & -3.4 \times 10^{-8} & 3.7 \times 10^{-8} & 2.25 \times 10^{-5} \\ 1.8 \times 10^{-5} & -3.4 \times 10^{-8} & 1.7 \times 10^{-7} & -2.4 \times 10^{-8} & -2.7 \times 10^{-5} \\ 3.0 \times 10^{-6} & 3.7 \times 10^{-8} & -2.4 \times 10^{-8} & 3.0 \times 10^{-8} & -1.3 \times 10^{-5} \\ 0.05 & 2.2 \times 10^{-5} & -2.7 \times 10^{-5} & -1.3 \times 10^{-5} & 0.38 \end{bmatrix}.$$

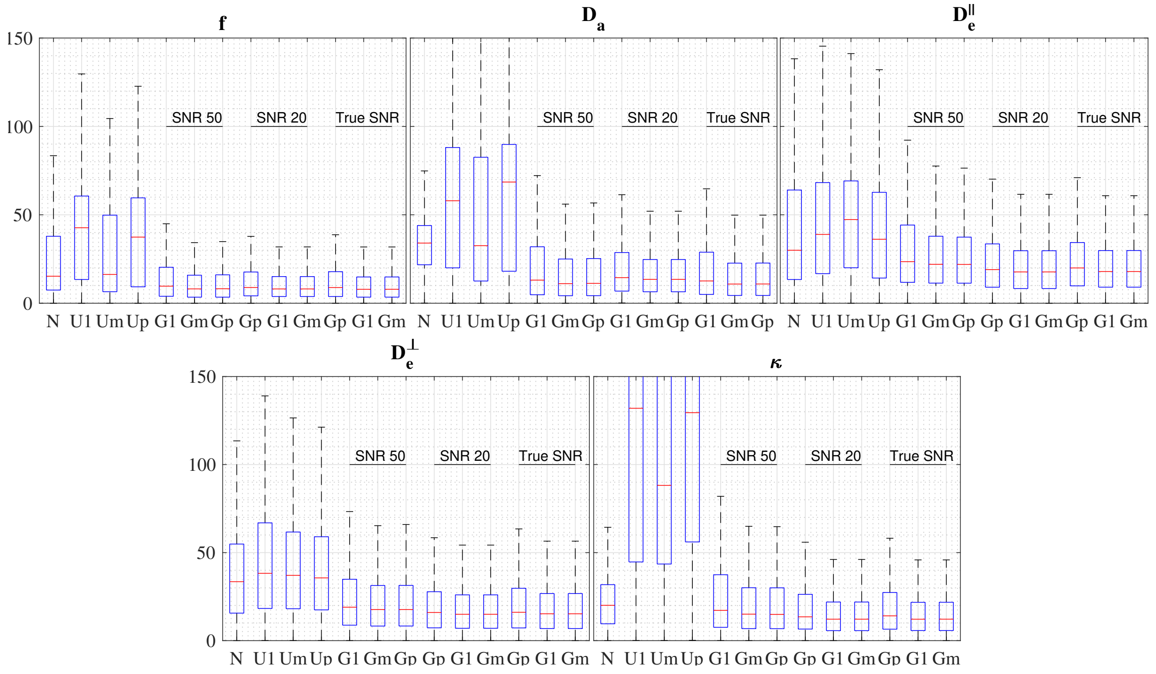

Supporting Information Figure S2: Accuracy of the estimation of the NODDIDA parameters by each of the previously proposed estimation methods (N, U1, Um, Up) and G1, Gm, Gp assuming SNR 50, assuming SNR 20, and using their true (calculated) SNRs. The boxplots represent the distribution of the relative estimation error for each NODDIDA parameter and for each method, with a clinically fesible subset of measurements. We observe a slight improvement in the estimation of $\kappa, D_e^{\|}$ and $D_e^{\perp}$, using the proposed methods, when SNR 20 or the estimated SNRs were used. This is because, the mean SNR from all the MGH HCP data was around 35, and hence choosing SNR 20 was a closer approximation to the true SNR, than choosing SNR 50.

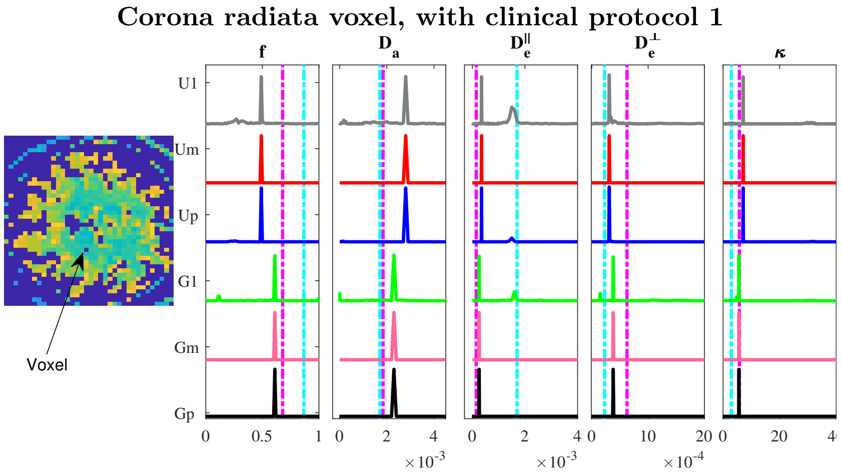

Supporting Information Figure S3: Estimates from a voxel of corona radiata with clinical subset of measurements. The cyan dotted lines represent the estimates using NODDI. The parameter estimates using the full extended protocol (GT) is shown in magenta dotted lines.

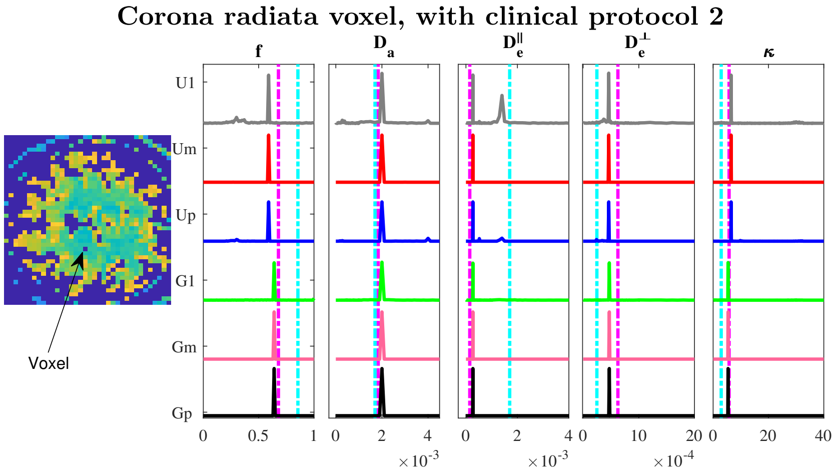

Supporting Information Figure S4: Estimates from a voxel of corona radiata with a different subset of measurements. The cyan dotted lines represent the estimates using NODDI. The parameter estimates using the full extended protocol (GT) is shown in magenta dotted lines.

It can be seen that using clinical protocol 2, we obtain estimates closer to the GT, compared to clinical protocol 1, with uniform prior. The proposed population-based prior gave better estimates, closer to the GT, for both protocols. We believe this is because clinically feasible measurements, are insufficient to capture the complexity of water diffusion in corona radiata, which has a complex orientation. Thus we obtain incorrect estimates for certain clinically feasible protocols. The prior information in the proposed population-based prior thus aids the estimation of NODDIDA parameters to obtain better estimates.

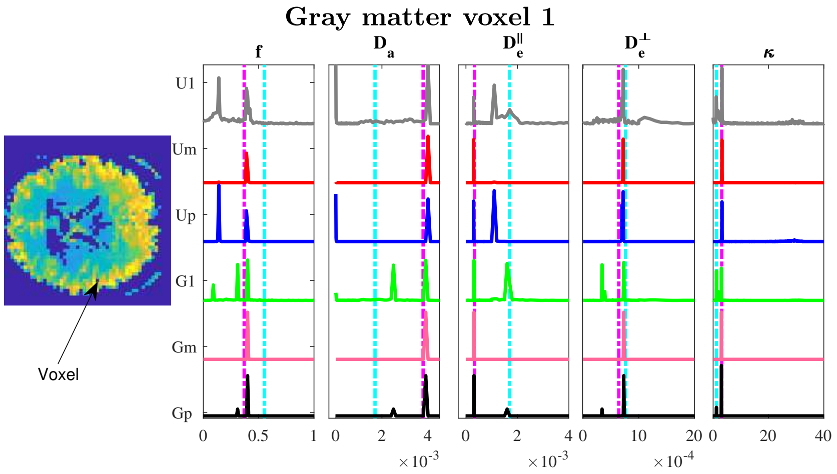

Supporting Information Figure S5: Estimates from a gray matter voxel with clinical subset of measurements. The cyan dotted lines represent the estimates using NODDI. The parameter estimates using the full extended protocol (GT) is shown in magenta dotted lines.

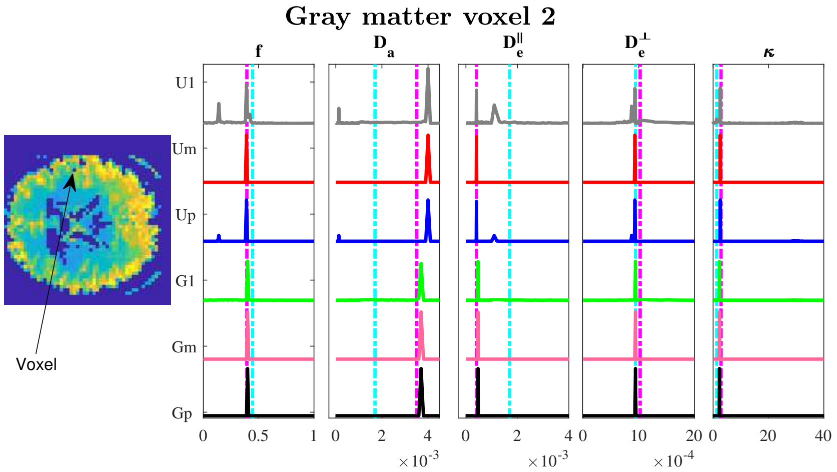

Supporting Information Figure S6: Estimates from a different gray matter voxel with clinical subset of measurements. The cyan dotted lines represent the estimates using NODDI. The parameter estimates using the full extended protocol (GT) is shown in magenta dotted lines.

Both these voxels demonstrate relatively high diffusion (parameters $D_a, D_e^{||}, D_e^{\perp}$), and low orientation distribution parameter κ, compared to white matter, as reported earlier. The proposed prior provides better estimates compared to the uniform prior.
